# One Woman, One Nation: The Heroic Story of Dr. Stella Adadevoh

**DOI:** 10.7759/cureus.71650

**Published:** 2024-10-16

**Authors:** Muhammed Raji Modibbo, Hadiza Ibrahim, Fatima S Ahmed

**Affiliations:** 1 Internal Medicine, Federal Medical Centre, Abuja, NGA; 2 Internal Medicine, Zayed Military Hospital, Abu Dhabi, ARE; 3 General Practice, Duchess International Hospital, Lagos, NGA

**Keywords:** ebola, ebola virus, ebola virus disease epidemic, ethic, ethical and legal principles in medical practice, lagos, nigeria

## Abstract

In 2014, Nigeria faced a near-existential threat when Ebola was introduced to Lagos, a city of over 20 million people. Dr. Stella Ameyo Adadevoh (1956-2014), a physician at First Consultant Hospital, played a pivotal role in preventing the spread of the virus by making the crucial decision to quarantine the index case, Patrick Sawyer, despite intense governmental pressure to release him. This review explores Dr. Adadevoh’s life, her heroic actions during the Ebola crisis, and the lasting impact of her work on Nigeria’s public health system. It also highlights the ethical and professional challenges she faced, including balancing patient autonomy with the need for public safety. Through her brave decision-making, Dr. Adadevoh sacrificed herself to save countless lives and ensured Nigeria’s swift containment of the deadly virus, leaving behind an inspirational legacy of medical ethics, leadership, and courage that continues to influence public health policy today. This paper highlights the lessons learned from her actions, emphasizing the critical role of decisive leadership in managing infectious disease outbreaks and the profound selflessness and courage she demonstrated.

## Introduction and background

**Figure 1:**
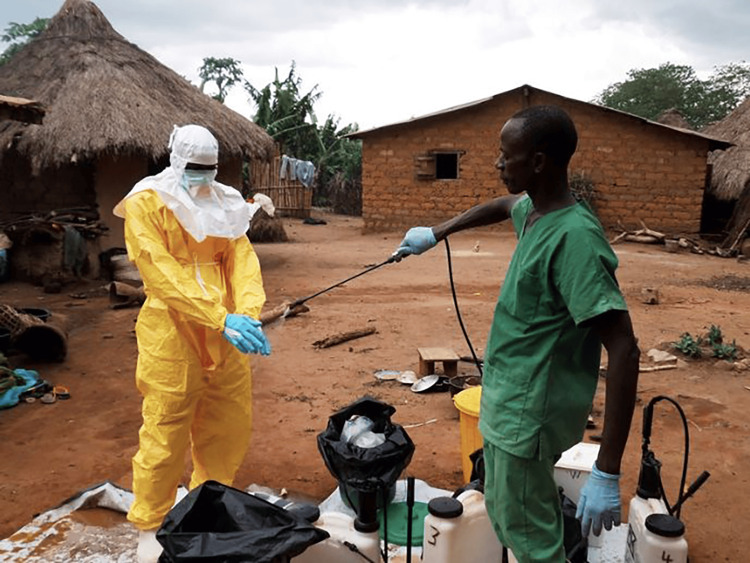
The fight against Ebola in West Africa. Image © 2015 EC/ECHO/Jean-Louis Mosser, licensed under the Creative Commons Attribution-NonCommercial-NoDerivs 2.0 License. Full license details are available at [9].

Ebola is a severe and potentially fatal viral illness [1]. It is caused by the Ebola virus, from the filovirus family, and is often spread through direct human contact, contact with infected bodily fluids, and contact with contaminated fomites [1]. Additionally, it has been well-documented that Ebola can be transmitted to humans through contact with wild animals hunted for food [2] and exposure to fruit bats [2,3]. Nearly 50% of people who contract Ebola die from the disease [4]. However, the death rate can vary significantly depending on the specific outbreak and control measures implemented, with rates ranging from as low as 25% to as high as 90% [4]. The condition usually manifests acutely with initial symptoms of fever, fatigue, myalgia, headache, and sore throat [1]. These symptoms are often quickly followed by vomiting, diarrhea, rash, and possibly internal and external hemorrhage, leading to multi-organ dysfunction [1,4]. The virus was first identified in 1976 and has since caused many outbreaks in various areas of sub-Saharan Africa [5]. The largest outbreak occurred in West Africa from 2013 to 2016 (Figure 1), resulting in 11,000 official deaths [5]. The epidemic began in December 2013 in a small village in Guinea after a young child and his family succumbed to a brief illness. The infection quickly spread across the subcontinent, devastating multiple countries and triggering a global health crisis [6-8].

On July 20, 2014, a very sick traveler from Liberia arrived at the International Airport in Lagos, Nigeria [10]. Soon after landing, he was admitted and diagnosed with Ebola virus disease [10], marking the first confirmed case of Ebola in the country. At the time, Nigeria's physician-to-population ratio was approximately 0.4 physicians per 1,000 people [11], significantly below the WHO's recommended standard. This deficit highlighted the challenges within the country's healthcare system, indicating that it would have struggled to effectively manage a raging epidemic like Ebola. The limited number of healthcare professionals would hinder timely diagnosis, treatment, and containment efforts, coupled with healthcare accessibility issues, the nation was not ready to respond adequately to such a public health crisis. Dr. Ameyo Stella Adadevoh, a Consultant Endocrinologist and Physician, assessed the patient and made the correct diagnosis [12]. Dr. Adadevoh faced an ethical dilemma in balancing patient autonomy with public safety when she had to decide whether to respect an Ebola patient's wishes to leave the hospital or to prioritize the health of the broader community by keeping the patient under quarantine. She took it upon herself to keep the patient against his wishes and against the insistence of the Liberian ambassador, who even threatened legal action. Dr. Adadevoh did not waver. 'For the greater public good,' she intoned [12,13]. After the diagnosis was confirmed, the Federal Ministry of Health was quickly alerted to the index case, and emergency measures were initiated. The Nigerian government’s response was both swift and effective. The protocols implemented meant that the spread of Ebola in Nigeria was curtailed to 20 confirmed and probable cases [10]. On October 20, 2014, the WHO declared Nigeria Ebola-free [14]. Unfortunately, Dr. Adadevoh lost her life to this virus, passing away on August 19, 2014 [15]. Nigeria was one of the very few countries in the subregion that was able to contain this epidemic, and it would not have been possible without the expertise, courage, and moral strength of its heroine. The objectives of this paper are to highlight the invaluable lessons that the younger generation can draw from her life: courage, ethical responsibility, clinical acumen, and the importance of standing firm in the face of adversity.

## Review

Early life and medical career

**Figure 2:**
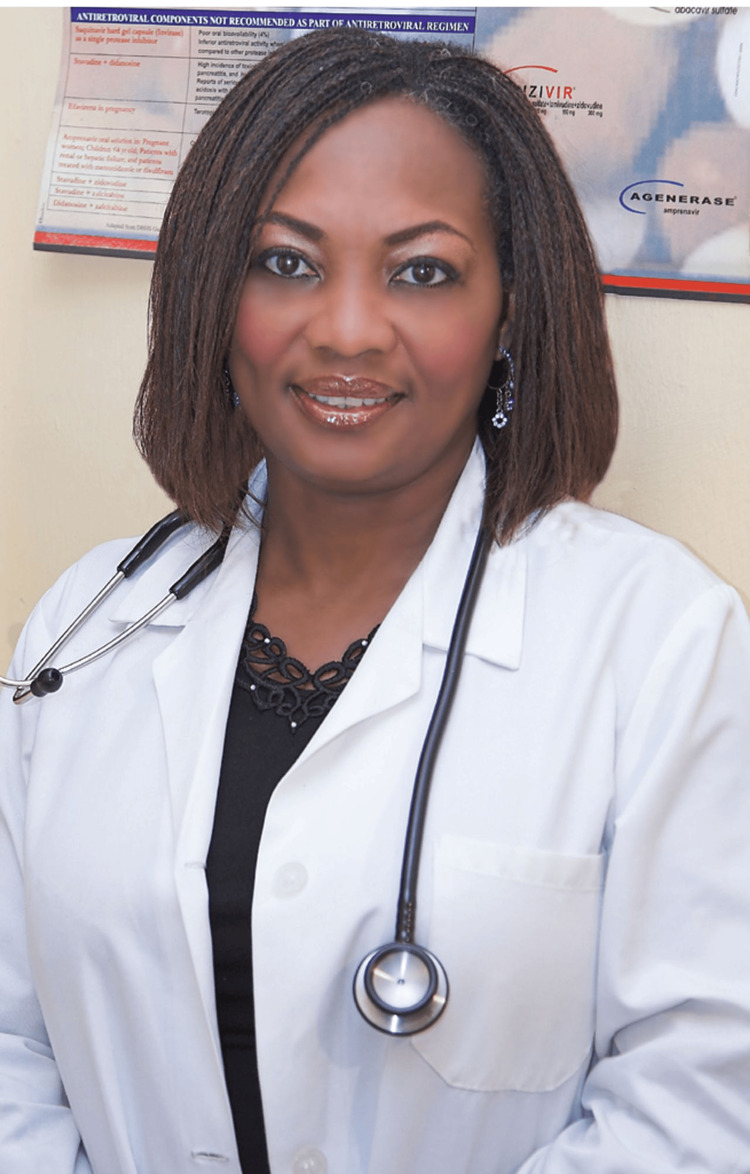
Dr. Ameyo Stella Adadevoh. Image courtesy of Dr. Ameyo Stella Adadevoh Health Trust (DRASA), used with permission.

Dr. Adadevoh (Figure 2) was born in October 1956 in Lagos, where she also grew up [16]. She came from a lineage of significant political and historical Nigerian figures. Her great-grandfather was the late Herbert Macaulay, who is widely regarded as one of the most prominent founders of modern Nigeria [12]. She was also the grand-niece of Nigeria’s first democratically elected president, Dr. Nnamdi Azikiwe [12]. Her father was also a renowned physician and academic, serving as a consultant and advisor to numerous international organizations such as the WHO and several United Nations agencies and commissions [12].

At the age of 24, she qualified as a medical doctor with an MBBS degree from the University of Lagos [17-19]. After medical school, Dr. Adadevoh completed her internship at Lagos University Teaching Hospital (LUTH) and subsequently fulfilled her National Youth Service Corps assignment at the Eti-Osa Health Centre in Lagos [17-19]. Soon after, she began her residency training at LUTH, which spanned from 1983 to 1988, after which she became a member of the West African College of Physicians. Following her residency, Dr. Adadevoh worked as a consultant at LUTH until 1991, when she earned a prestigious British Council Scholarship that allowed her to pursue a fellowship in Endocrinology at Hammersmith Hospital, Imperial College, London. Upon returning to Nigeria, she practiced for more than 20 years. She joined First Consultants Medical Centre (FCMC), Lagos, where she became the Lead Consultant Physician and Endocrinologist [17-19]. In 2012, there was an outbreak of H1N1 (swine flu) in Lagos, and Dr. Adadevoh was the first physician to diagnose and alert the Ministry of Health [19].

Ebola in Nigeria

In July 2014, despite the likelihood of an Ebola infection, Mr. Patrick Sawyer, a Liberian diplomat, was permitted to travel to Nigeria [20]. Following confirmed contact with his sister, who had succumbed to the illness, his employer had already suspended him from work and referred him to the Liberian Ministry of Health for the mandated isolation period [20]. However, Sawyer was granted clearance to attend a meeting of the Economic Community of West African States (ECOWAS) in Nigeria [20]. He entered Lagos on July 20, after leaving quarantine in Liberia [19]. He collapsed at the airport in Lagos and was taken to FCMC, a private hospital in the state [19]. The first doctor who attended to Mr. Sawyer made an initial assessment of malaria. Dr. Adadevoh did not agree with the initial diagnosis and suspected a case of Ebola virus disease [19]. Mr. Sawyer vehemently denied any recent sick contact, but Dr. Adadevoh contacted the government and insisted on testing him [19]. While investigations were still pending, Mr. Sawyer and other Liberian government officials began insisting that he be discharged to attend the ECOWAS conference. The Liberian Ambassador became involved, and immense pressure was placed on FCMC and Dr. Adadevoh to release Mr. Sawyer, with threats of sanctions and legal action [12,19]. Mr. Sawyer was livid about being kept in the hospital; eyewitness accounts report that he pulled out his IV cannula, causing spillage of blood all over his room [13].

"The only way we could be sure and live up to our responsibility to our people, the state, and nation, this is all about patriotism at the end of the day, was to keep him here," Dr. Adadevoh and the hospital resolved not to let him leave isolation [12,13]. After the diagnosis was made, the Nigerian government quickly declared an Ebola emergency and established the Emergency Operations Center (EOC) to manage the outbreak [10]. Despite the high population density and the risk of rapid transmission in Lagos, a coordinated rapid response helped contain the virus. The EOC, with international aid, played an instrumental part in containing the outbreak [10]. Early contact tracing efforts were initiated, and the case management team isolated patients and decontaminated affected areas. As of late September, 891 contacts had exited follow-up, and no new cases had been reported, suggesting the outbreak was under control [10]. Before Mr. Sawyer, Nigeria had never seen an Ebola case, so it was a remarkable feat of diagnostic expertise on Dr. Adadevoh’s part. And while protecting the country, Dr. Adadevoh and the other healthcare workers were themselves at grave risk; she contracted Ebola and died alongside three of her colleagues. Her incredible actions prevented a major outbreak and national catastrophe in a country of nearly 200 million people [12]. Dr. Adadevoh’s courage, clinical acumen, and confident leadership allowed the government to pull the resources needed to deal with Ebola. She facilitated a tactical curtailment of the virus across the country, and the Nigerian government was able to successfully trace all potential contacts, including all 20 cases, from that single path of transmission, originating from the index patient [12]. Because of her, Nigeria fared much better than most of its next-door neighbors that were ravaged by the epidemic.

Ethical and legal lines

Involuntary isolation and quarantine are public health tools used to manage contagious diseases, aiming to protect the wider population. However, forced isolation raises complex ethical and legal issues regarding patient liberty and autonomy [21]. Dr. Adadevoh's decision during the Ebola outbreak highlights this dilemma. It exemplifies the delicate balance between individual rights and the need to protect the broader community, underscoring the challenges inherent in such situations. According to the WHO, detaining patients against their will may sometimes be legally justified in cases where they pose a significant and immediate threat to public health and safety, such as with highly contagious diseases like multi-drug resistant tuberculosis [22]. In February 2021, Nigeria’s House of Representatives introduced a bill granting health officers the authority to detain individuals who fail to comply with isolation requirements, but emphasized that detention must be carried out by the least restrictive means available [23]. These provisions aim to ensure public health safety while minimizing infringement on individual liberties.

Legacy 

**Figure 3:**
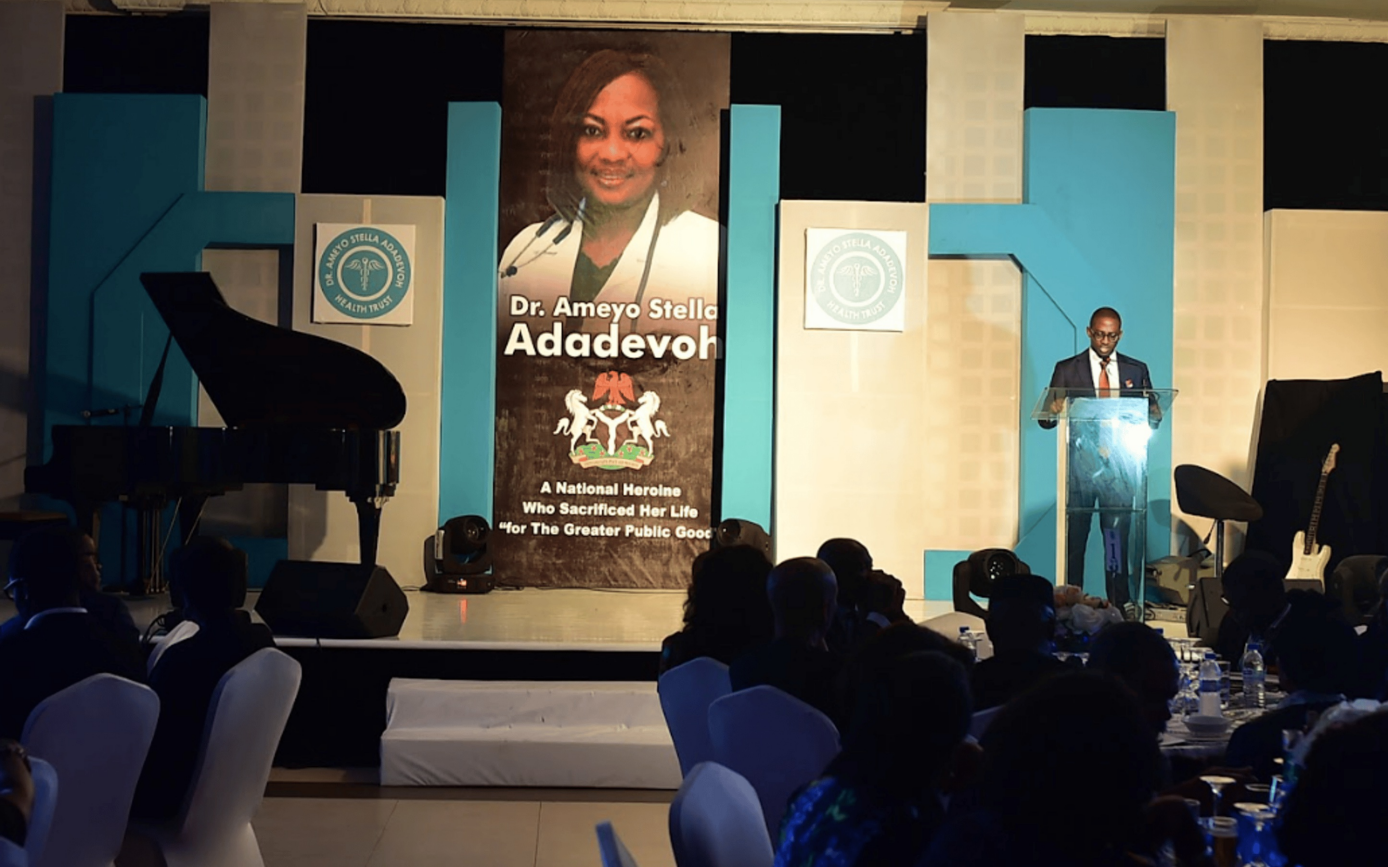
Launching Event of Dr. Ameyo Stella Adadevoh Health Trust (DRASA). Image courtesy of Dr. Ameyo Stella Adadevoh Health Trust (DRASA), used with permission.

**Figure 4:**
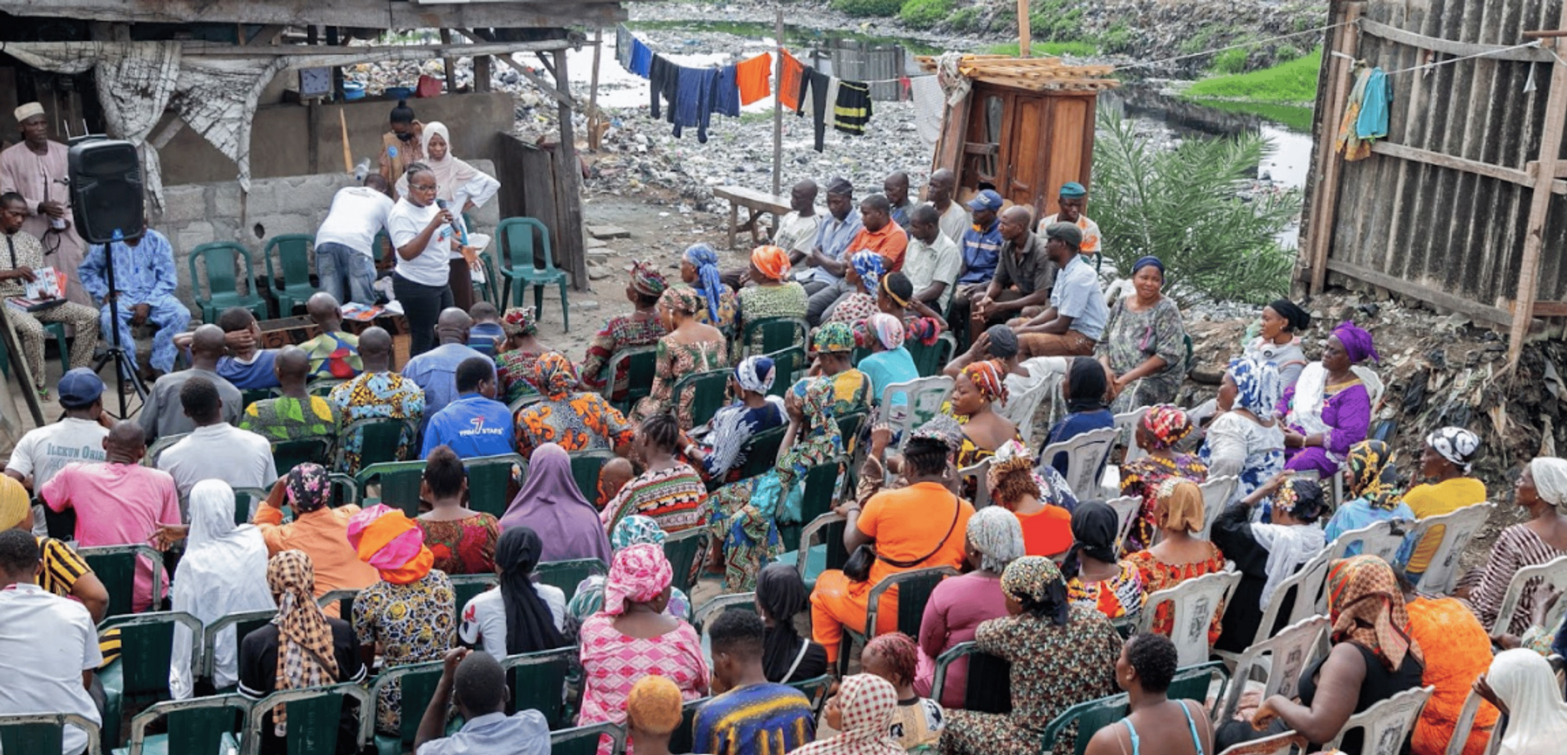
Health promoting community engagement by DRASA. Image courtesy of Dr. Ameyo Stella Adadevoh Health Trust (DRASA), used with permission.

One of her greatest legacies is the Dr. Ameyo Adadevoh Health Trust (DRASA), a nonprofit public health organization created in her memory (Figure 3) [24]. DRASA focuses on building a resilient and accessible health system, grounded in the belief that one person’s actions can inspire widespread change. The organization aims to reduce the spread of infectious diseases and help save lives. Their vision is a healthy society supported by a robust health system and well-equipped health champions to mitigate preventable infections [24]. So far, DRASA has engaged over 56,000 beneficiaries through capacity-building programs, empowering communities (Figure 4) with essential public health knowledge [25]. They have distributed over 31,000 PPE clothing and equipment to protect health workers and prevent disease transmission between them, patients, and the environment [25]. Additionally, over 3,200 teachers and school staff have been trained to ensure student safety and promote a healthy school environment [25]. Beyond these achievements, DRASA has made significant strides in other areas, including engaging and educating community leaders, teaching students proper hygiene and health practices, empowering border and security officers to manage sudden outbreaks, and addressing numerous other health and wellness indices [25].

Additionally, the Netflix Original film "93 Days" is dedicated to Dr. Adadevoh and depicts the heroic efforts of her and the medical team at First Consultant Medical Center in treating Patrick Sawyer during the Ebola outbreak in Nigeria [26]. The film serves as a powerful tribute to her bravery and sacrifice, helping to preserve her memory and educate the public about her contributions to public health. On what would have been her 62nd birthday, she was honored with a special Google Doodle in memoriam on October 27, 2018, reflecting a lasting recognition of her contributions and the mark she left on the world [27]. In February 2020, a road in the capital city of Nigeria was named "Ameyo Adadevoh Way" in her honor [28]. This naming was among the Nigerian government's initial efforts to recognize her significant contributions. Additionally, CNN honored her as one of the Most Inspiring Women of 2014, according to Leading Women. This recognition highlights her as one of the exceptional women who have achieved significant success in their respective fields [29]. And in October 2022, she was posthumously conferred with the National Honor of Officer of the Order of Niger (OON) by His Excellency President Muhammadu Buhari, a prestigious accolade recognizing her tremendous contributions to the nation [30].

Above all, however, her greatest legacy is her exceptional diagnostic skills and her ability to maintain a high index of suspicion, particularly for the younger generation of healthcare professionals. Her clinical acumen, which enabled her to diagnose Ebola quickly in a high-stakes situation, underscores the importance of remaining vigilant and thorough, even when facing unexpected or rare conditions. This practice of keeping a heightened awareness can save lives, especially in infectious disease management. Beyond her clinical expertise, Dr. Adadevoh also exemplified the courage to stand firm in her decisions despite external pressures. Her refusal to release an infected patient, even under threats of legal action, serves as a powerful reminder to always prioritize the greater good when you know you are doing the right thing. These lessons in clinical excellence and moral courage are essential for young professionals who must often make difficult decisions under pressure.

## Conclusions

Dr. Stella Ameyo Adadevoh's legacy is a profound testament to unwavering dedication and moral courage. Her decisive actions during the Ebola outbreak in Nigeria not only averted a national disaster but also highlighted the vital role of strict infection control and the immense value of healthcare workers who act with integrity and selflessness. Her story serves as a beacon, emphasizing the importance of preparedness, vigilance, and compassion in healthcare systems worldwide. It also reinforces the need for continued investment in healthcare infrastructure and the nurturing of professionals who are prepared to face both expected and unexpected challenges. As we reflect on her remarkable sacrifice, we are reminded of the critical role medical professionals play in safeguarding public health, especially in times of crisis. In her courage, selflessness, and unwavering love for her country, Dr. Adadevoh reflected the vision our founding fathers had for a united and thriving nation. Her sacrifice stands as a powerful reminder of what it means to truly serve one’s nation, showing us that the Nigerian Dream is alive in those who, like her, value the well-being of others above personal gain.
